# Adeno-associated virus 2 CRISPR/Cas9-mediated targeting of hepatitis B virus in tree shrews

**DOI:** 10.1016/j.virusres.2025.199550

**Published:** 2025-02-21

**Authors:** Md Haroon Or Rashid, Mohammad Enamul Hoque Kayesh, Md Abul Hashem, Tatsuro Hifumi, Shintaro Ogawa, Noriaki Miyoshi, Yasuhito Tanaka, Michinori Kohara, Kyoko Tsukiyama-Kohara

**Affiliations:** a Laboratory of Animal Hygiene, Joint Faculty of Veterinary Medicine, Kagoshima University, Kagoshima, Japan; b Transboundary Animal Diseases Centre, Joint Faculty of Veterinary Medicine, Kagoshima University, Kagoshima, Japan; c Department of Microbiology and Public Health, Patuakhali Science and Technology University, Bangladesh; d Department of Cell and Developmental Biology, Feinberg School of Medicine, Northwestern University, Chicago, IL, USA; e Department of Veterinary Histopathology, Joint Faculty of Veterinary Medicine, Kagoshima University, Kagoshima, Japan; f Faculty of Life Sciences, Kumamoto University 1-1-1 Honjo, Chuo-ku, Kumamoto, Japan; g Department of Microbiology and Cell Biology, Tokyo Metropolitan Institute of Medical Science, Japan

**Keywords:** Hepatitis B virus, HBV-F, CRISPR/Cas9, Adeno-associated virus 2, Northern tree shrew, Tupaia

## Abstract

Chronic hepatitis B virus (HBV) infection is a global health issue with limited therapeutic options given the persistence of viral episomal DNA (cccDNA). Previously, we investigated the effects of adeno-associated virus 2 (AAV2) vector-mediated delivery of three guide (g)RNAs/Cas9 selected from 16 gRNAs. AAV2/WJ11-Cas9 effectively suppressed HBV replication *in vitro* and in humanized chimeric mouse livers. In the present study, we examined the effect of AAV2/WJ11-Cas9 on the acute phase of HBV genotype F infection in an immunocompetent northern tree shrew (*Tupaia belangeri*; hereafter, “tupaia”) model. AAV2/WJ11-Cas9 treatment significantly reduced the HBV viral load in serum at 1, 7, 10, and 14 days post-infection (dpi). HBV-F infection caused enlargement of hepatocytes and mild lymphocytic infiltration in the interlobular connective tissue. Thus, the virus damages hepatocytes and drives infection progression and HBV core antigen (HBcAg) accumulation, which were not observed in AAV2/WJ11-Cas9 treated and normal liver tissues. AAV2/WJ11-Cas9 treatment reduced HBV DNA and cccDNA in liver tissues, as well as serum levels of HBV surface antigen and HBV core-related antigen (HBcrAg), including HBcAg and HBeAg at 14 dpi. Anti-HBc, anti-HBs, and anti-AAV Abs production was also detected. AAV2/WJ11-Cas9 treatment suppressed inflammatory cytokines and *TLR1, TLR2, TLR3, TLR4, TLR6, TLR7*, and *TLR9* mRNA levels. Thus, WJ11/Cas9 delivered by AAV2 vectors may provide a new therapeutic approach for inhibiting HBV infection in immunocompetent animal models, which could be developed for use in humans through further translational research.

## Highlights

• AAV2/WJ11-Cas9 reduced HBV-F DNA and cccDNA in the tupaia liver and sera.
• Histological recovery was observed in AAV2/WJ11-Cas9-treated tupaia.
• Cytokines and TLR suppression suggest anti-inflammatory effect of AAV2/WJ11-Cas9.

## 1 Introduction

Hepatitis B virus (HBV), a member of the family *Hepadnaviridae*, is an enveloped, circular, and partially double-stranded DNA virus that constitutes a major cause of chronic hepatitis, liver cirrhosis, and hepatocellular carcinoma (El-Serag, 2012; Li et al., 2020). HBV has eight well-known genotypes (A–H), with >8 % difference in the nucleotide sequence of the genome and a distinct geographic distribution (Arauz-Ruiz et al., 2002; Bello et al., 2023; Marciano et al., 2013; Okamoto et al., 1988). Over 254 million people are chronically infected with HBV worldwide with 1.2 million new infections occurring annually. Current therapies with nucleoside/nucleotide analogs can suppress viral replication by attenuating the activity of viral reverse transcriptase (Yuen et al., 2018); however, they are unable to eliminate replicative HBV templates comprising covalently closed circular DNA (cccDNA) or integrated HBV DNA (Li et al., 2017). Moreover, integrated HBV DNA contributes to HBsAg production (Meier et al., 2021) and is associated with carcinogenesis (Pollicino et al., 2019; Qian et al., 2024). Therefore, the eradication of persistent HBV cccDNA or integrated HBV DNA from infected cells is crucial to achieve a complete cure for chronic HBV infection (Koumbi, 2015; Yardeni et al., 2023), highlighting the need to explore alternative therapeutic approaches to eliminate this residual HBV DNA.

Clustered regularly interspaced short palindromic repeats (CRISPR)-associated protein 9 (Cas9) is a genome editing tool that has shown potential as a therapeutic approach against viral infections (Bartosh et al., 2023; Cai et al., 2023; White et al., 2015). Within this system, tools for delivering Cas9 and guide RNA (gRNA) are key factors influencing its efficiency. Adeno-associated virus (AAV), a nonpathogenic parvovirus, has become one of the most promising viral vectors for human gene therapy (Shen et al., 2007). In particular, AAV2-based vectors may offer a flexible delivery method for CRISPR/Cas9 gene editing. We previously investigated the effects of AAV2 vector-mediated delivery of three gRNAs/Cas9 genes selected from 16 gRNAs (Kayesh et al., 2020). These significantly suppressed HBV replication in cells, with WJ11/Cas9 exhibiting the highest efficacy, and being chosen for *in vivo* studies. AAV2/WJ11-Cas9 also significantly inhibited HBV replication and reduced cccDNA levels in the tested cells. Moreover, AAV2/WJ11-Cas9 enhanced the effects of entecavir when used in combination, indicating a different mode of action. Notably, in humanized chimeric mice, AAV2/WJ11-Cas9 significantly suppressed HBcAg, HBsAg, and HBV DNA and cccDNA in liver tissues without significant cytotoxicity; next generation sequencing data showed no significant genomic mutations (Kayesh et al., 2020).

Although we confirmed the anti-HBV effect of AAV2/WJ11-Cas9 *in vitro* and *in vivo*, its efficacy needs to be further analyzed in immunocompetent animal models. Previously, chimpanzees have been used as natural infection models for HBV; however, high cost and ethical concerns restrict the experimental use of chimpanzees. Humanized chimeric mice (Mercer et al., 2001) are effective animal models of HBV infection (Nakagawa et al., 2013); however, characterization of the host immune response is not possible. To address this issue, we previously established a northern tree shrew (*Tupaia belangeri*; hereafter, “tupaia”) model (Tsukiyama-Kohara and Kohara, 2014) and have used it to study HBV infection (Kayesh et al., 2017a; Li et al., 2021; Sanada et al., 2019, 2016; Walter et al., 1996). HBV-F Mt (BCP/PC/2051), containing A1762T/G1764A, G1896A, and A2051C mutations, was observed in patients with HCC and could replicate more efficiently than HBV-F Wt (Hayashi et al., 2019). We also investigated the immune response in the tupaia model (Kayesh et al., 2017a). In the present study, we aimed to understand the effect of AAV2/WJ11-Cas9 on HBV infection and host immune responses using the HBV-F Mt tupaia model.

## 2 Materials and methods

### 2.1 Ethics statement

This study was conducted in strict accordance with the Guidelines for Animal Experimentation of the Japanese Association for Laboratory Animal Science, and the recommendations of the Guide for the Care and Use of Laboratory Animals of the National Institutes of Health. All protocols for the tupaia experiment were approved by the regional ethics committee (VM13044).

### 2.2 Animals

Northern tree shrews (*Tupaia belangeri*) were purchased from the Kunming Institute of Zoology, Chinese Academy of Sciences. The animals, aged 4 to 6 years (F1–F2), were born in the animal facilities of Kagoshima University, Japan. Nine tupaias were assigned to three groups: CRISPR/Cas9-treated, Mock-treated, and Normal (*n* = 3). The Normal group consisted of tupaias that were not infected with HBV and were used for comparative histopathological and cytokine expression analyses.

### 2.3 Plasmids

The AAVpro CRISPR/Cas9 Helper-Free System (AAV2) (Clontech Laboratories, Inc., Palo Alto, CA, USA) was used to construct the gRNA/Cas9 expression plasmids, and AAV2 particles were prepared as described previously (Kayesh et al., 2020). The gRNA sequence of WJ-11 (positions 1859–1878) used in this study was ACTGTTCAAGCCTCCAAGCT.

### 2.4 Production and purification of AAV2 particles

For the preparation of AAV2-Guide-it-Up and AAV2-Guide-it-Down-gRNA vectors (purchased from TaKaRa Bio Inc., Shiga, Japan), HEK293 cells were transiently transfected using the Xfect Transfection Reagent (Clontech Laboratories, Inc.) according to the manufacturer's instructions. Briefly, HEK293 cells were co-transfected with the pAAV-Guide-it-Up or pAAV-Guide-it-Down vector expression plasmid, an AAV2 ITR-containing plasmid carrying the Cas9/gRNA genes, a pRC2-mi342 plasmid expressing AAV2 Rep and Cap proteins, and pHelper plasmid expressing adenovirus helper proteins (Clontech Laboratories, Inc.). To accommodate the *Streptococcus pyogenes* Cas9, which is >4 kb in length, a dual AAV platform consisting of either the pAAV-Guide-it-Up or pAAV-Guide-it-Down vector was used. At 72 h post-transfection, cells were collected, and AAV2 particles were harvested using AAV extraction solutions A and B (Clontech Laboratories, Inc.) according to the manufacturer's instructions. The harvested AAV2 particles were stored at −80 °C for further use. SignaGen Laboratories prepared the AAV2 particles to a large scale (10^13^ copies) for *in vivo* experiments.

AAV2 vectors were purified using an AAVpro Purification Kit (all serotypes) (TaKaRa Bio Inc.) according to the manufacturer's instructions. After purification, the viral titer (viral genomic titer) was determined through real-time polymerase chain reaction (PCR) targeting the ITR domain, using an AAVpro Titration Kit (TaKaRa Bio Inc.), following the manufacturer's instructions.

### 2.5 Inoculation of AAV2/WJ11-Cas9 to HBV-infected tupaias

To examine the anti-HBV effects of the HBV-specific AAV2/WJ11-Cas9 system *in vivo*, six adult tupaias infected with HBV genotype F mutant strain (GenBank LC831106.1) were used, with three tupaias used as non-treated controls (marked as 178, 191, and 386) and the others inoculated with AAV2/WJ11-Cas9 (marked as 323, 380, and 329). HBV genotype F (10^7^ copies) was administered intravenously (iv) (Mercer et al., 2001) simultaneously with AAV2/WJ11 or mock treatment. A total of 5 × 10^12^ vg copies of AAV2/WJ11-Cas9 or an equal volume of sterile Opti-MEM (non-treated control) were injected through the tail vein. Tupaia serum was collected on days −4, 1, 3, 5, 7, 10, and 14 post-inoculation. Serum HBV DNA titers, along with alanine aminotransferase (ALT) were measured at each indicated time point. Fourteen days post-inoculation, the tupaias were sacrificed under anesthesia after whole blood collection, and tissue samples were collected and stored at −80 °C for further characterization or placed in 10 % formaldehyde-PBS (WAKO) for histological examination (hematoxylin and eosin staining). Liver tissue was also applied for HBcAg detection, as described previously (Kayesh et al., 2020).

Intrahepatic HBV DNA and HBV cccDNA titers were determined from genomic DNA extracted from tupaia liver tissues using the phenol-chloroform extraction method. Serum ALT levels were determined using a Transnase Nissui kit (Nissui Pharmaceutical Co., Ltd.), and the data were standardized and are presented as IU/l.

### 2.6 HBV total DNA, cccDNA extraction and quantitation, and T7E1 assay

Total DNA was isolated from tupaia liver tissues as previously described (Kayesh et al., 2017a). To purify HBV cccDNA, total DNA was treated with Plasmid-Safe ATP-Dependent DNase (Zhong et al., 2014) (Lucigen Corporation) at 37 °C for 60 min, followed by DNase inactivation at 70 °C for 30 min. The digested products were used as templates for real-time PCR to quantify HBV cccDNA, as described below. The T7E1 assay was performed as previously described (Kayesh et al., 2017b; Liu et al., 2015). The primers used were completely matched to HBV-Fmt sequence; 1st PCR sense (794) 5ʹ-CCGCTGTTACCAATTTTCTGTTATC-3ʹ, 1st PCR anti-sense (2051) 5ʹ-GAGGTGTGCAATGTTCTGGTGATT-3ʹ, 2nd PCR sense 5ʹ-(819) TGTGGGTATCCATTTAAATA-3ʹ, and 2nd PCR anti-sense (2027) 5ʹ-CTAAAGCATCCCGGTAGAGG-3ʹ.

HBV DNA was quantified using real-time PCR as previously described (Cressman et al., 1996), with a detection limit of one copy of HBV DNA and HBV cccDNA per microgram of liver tissue. The primers and probes for the S gene included the forward primer HB-166-S21 (nucleotides (nts) 166–186): 5′-CAC ATC AGG ATT CCT AGG ACC-3′, the reverse primer HB-344-R20 (nts 344–325): 5′-AGG TTG GTG AGT GAT TGG AG-3′, and the TaqMan probe HB-242-S26FT (nts 242–267): 5′-CAG AGT CTA CAC TCG TGG ACT TC-3′.

HBV cccDNA quantification was performed using qPCR with TaqMan chemistry, using the forward primer HBVcccDNA 1519-S25 (5′-ACG GGG CGC ACC TCT CTT TAC GCG G-3′), the reverse primer HBVcccDNA 1886-R25 (5′-CAA GGC ACA GCT TGG AGG CTT GAA C-3′), and the Taq-Man probe HBVcccDNA-1575 S26FT (5′6-FAM–CCG TGT GCA CTT CGC TTC ACC TCT GC-TAMRA-3′). Cycling was performed as 50 °C for 2 min, 95 °C for 10 min, and 53 cycles at 95 °C for 20 s and 65 °C for 1 min. PCR was performed using a CFX Connect Real-Time PCR Detection System (Bio-Rad). Cytokines and TLR mRNAs were quantified via qRT-PCR using specific primers (Table S1), as previously reported (Kayesh et al., 2017a).

### 2.7 Measurement of HBsAg and HBcrAg

HBsAg levels in the tupaia serum was measured using the two-step sandwich assay principle with a fully automated chemiluminescent enzyme immunoassay system (Lumipulse G 1200, Fujirebio, Tokyo, Japan), as described previously (Shinkai et al., 2013). HBcrAg levels in tupaia serum were measured using a high-sensitivity HBcrAg assay, as described previously (Inoue et al., 2021).

### 2.8 Total RNA extraction from tupaia liver tissues and measurement of cytokines and TLR mRNA

Total RNA was extracted from tupaia liver tissues using the RNeasy Plus Mini Kit (QIAGEN) following the manufacturer's instructions, and genomic DNA was removed using the gDNA Eliminator Spin Column. The concentration and purity of the extracted RNA were measured using a NanoDrop ND-1000 spectrophotometer (NanoDrop Technologies, Inc., Waltham, MA, USA). The samples were stored at −80 °C until needed for gene expression analysis.

We analyzed the expression of TLR mRNAs (TLR1–9) in tupaia liver tissues using one-step qRT-PCR with Brilliant III Ultra-fast SYBR Green QRT-PCR Master Mix (Agilent Technologies, Santa Clara, CA, USA), following Kayesh et al. (2017a). The PCR process involved reverse transcription at 50 °C for 10 min, initial denaturation at 95 °C for 3 min, and 40 cycles at 95 °C for 5 s and 60 °C for 10 s. We also measured the expression of IL-6, TNF-α, IFN-γ, cGAS, and IFN-β mRNA using the same method, as described previously (Sanada et al., 2019; Kayesh et al., 2017a). The gene-specific primer sequences are provided in Table S1. The gene copy number per microgram of liver RNA was calculated using a standard curve from pre-quantified gene copies. Each sample was measured in duplicate, and statistical analysis was performed to compare with uninfected controls.

### 2.9 ELISA and immunoblot assay

Anti-HBc and anti-HBs antibodies in tupaia serum were detected via ELISA, as previously described (Ezzikouri et al., 2015) with slight modifications. Purified HBc and HBs antigens (Beacle Co., Osaka, Japan) were diluted with a sodium carbonate buffer (pH 9.0) (5 μg/mL) and a 1 % Block Ace solution (KAC Co. Ltd., Japan) in phosphate-buffered saline (-) containing 0.05 % Tween 20. This solution was used to block and dilute the antibodies. Tupaia serum and anti-tupaia IgG rabbit serum (in-house) were diluted 10,000-fold for reaction. Anti-HBc rabbit polyclonal antibody (ab115992, Abcam Co.) and anti-HBs mouse monoclonal antibody (MoAb) (MA1-19263, ThermoFisher) were used as positive controls.

Cas9 protein was detected in tupaia liver tissues via standard immunoblotting assay using an anti-Cas9 rabbit MoAb (#19,526, Cell Signaling Co.). Tissues were lysed in a lysis buffer (1 % SDS, 0.5 % NP40, 0.15 M NaCl, 10 mM Tris [pH 7.4], 5 mM EDTA, and 1 mM DTT). β actin was detected using an anti-β actin mouse MoAb (clone AC15, Merk Co.). Protein bands were visualized and quantified using a FUSION chemiluminescence imaging system (M&S Instruments, Inc.).

### 2.10 Statistical analysis

Data are presented as the means ± SD. For statistical analysis, Student's *t*-test for two groups and one-way ANOVA and Dunnett's multiple comparisons were performed using GraphPad Prism software. Statistical significance was set at *P* < 0.05.

## 3 Results

### 3.1 Anti-HBV effects of the AAV2/WJ11-Cas9 system

**Fig. 1:**
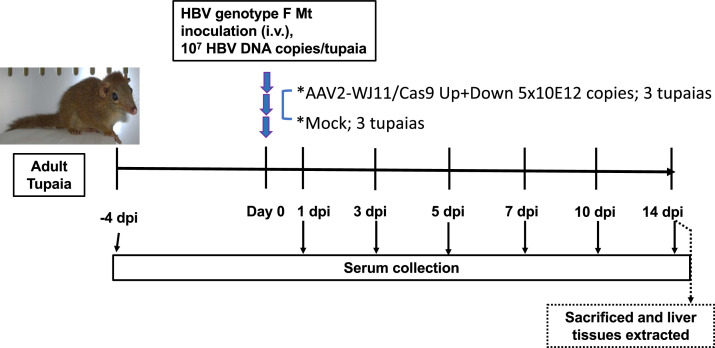
Experimental design of AAV2/WJ11-Cas9 and HBV genotype F mutant-type infection in adult tupaia. Adult tupaias (4–5 years) were either mock-treated or AAV2/WJ11-Cas9-treated, and HBV genotype F-Mt was simultaneously inoculated intravenously. Tupaia serum was collected −4, 1, 3, 5, 7, 10, and 14 days post infection (dpi).

**Fig. 2:**
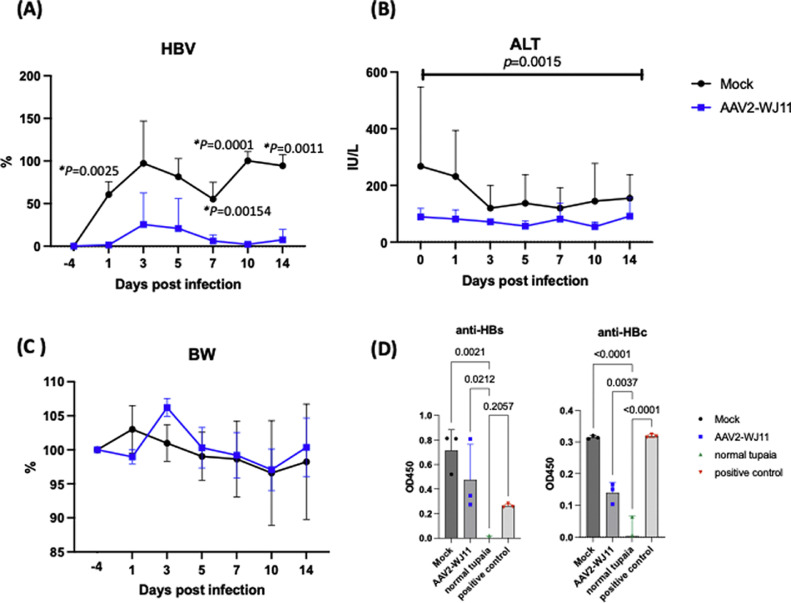
Effects of the AAV2/WJ11-Cas9 in the HBV-F-Mt-infected tupaia. (A) Detection of HBV DNA levels in serum at indicated time points. The level of HBV is indicated as a ratio to 10 dpi of HBV in mock-treated tupaia serum. A significant reduction in HBV levels was observed in AAV2/WJ11-Cas9-treated tupaia (*n* = 3) compared to that in non-treated controls (*n* = 3). (B) Serum ALT level, and (C) the ratio of body weight at indicated time points compared to day −4. (D) Anti-HBs and HBc antibodies were detected via ELISA in tupaia serum at 14 dpi with HBV infection. Error bars indicate the S.D. from three independent experiments. Statistical significance was calculated and p values <0.05 are indicated.

To examine the anti-HBV effects of the HBV-specific gRNA/Cas9 system *in vivo* in an immunocompetent HBV infection model, we injected AAV2/WJ11-Cas9 (5 × 10^12^ copies/tupaia) into the tail vein of a tupaia (Fig. 1). Simultaneously, HBV genotype F-Mt (10^7^ copies; entire nucleotide sequence is shown in GenBank LC831106.1) was injected intravenously as reported previously (Kayesh et al., 2017a). Blood samples were collected from tupaias on days −4, 1, 3, 5, 7, 10, and 14 days post HBV infection (dpi). We then measured HBV DNA and ALT levels in serum from mock-treated and AAV2/WJ11-Cas9-treated tupaias (Fig. 2). Significantly lower HBV DNA levels were observed in the serum of tupaia injected with AAV2/WJ11-Cas 9 than in mock-treated tupaia at 1, 7, 10, and 14 dpi (Fig. 2A). Administration of AAV2/WJ11-Cas9 did not yield any significant changes in ALT levels, although the overall ALT values in the mock-treated group were significantly higher than those in the AAV2/WJ11-Cas9-treated group (Fig. 2B). The peak of body weight in the mock-treated group was observed at 1 dpi (103 %), whereas the peak in the AAV2/WJ11-Cas9-treated group was observed at 3 dpi (106.3 %) (Fig. 2C). Anti-HBs and anti-HBc antibodies could be detected in tupaia serum via ELISA at 14 dpi (Fig. 2D).

### 3.2 Mock- and AAV2/WJ11-Cas9-treated tupaia liver tissues

**Fig. 3:**
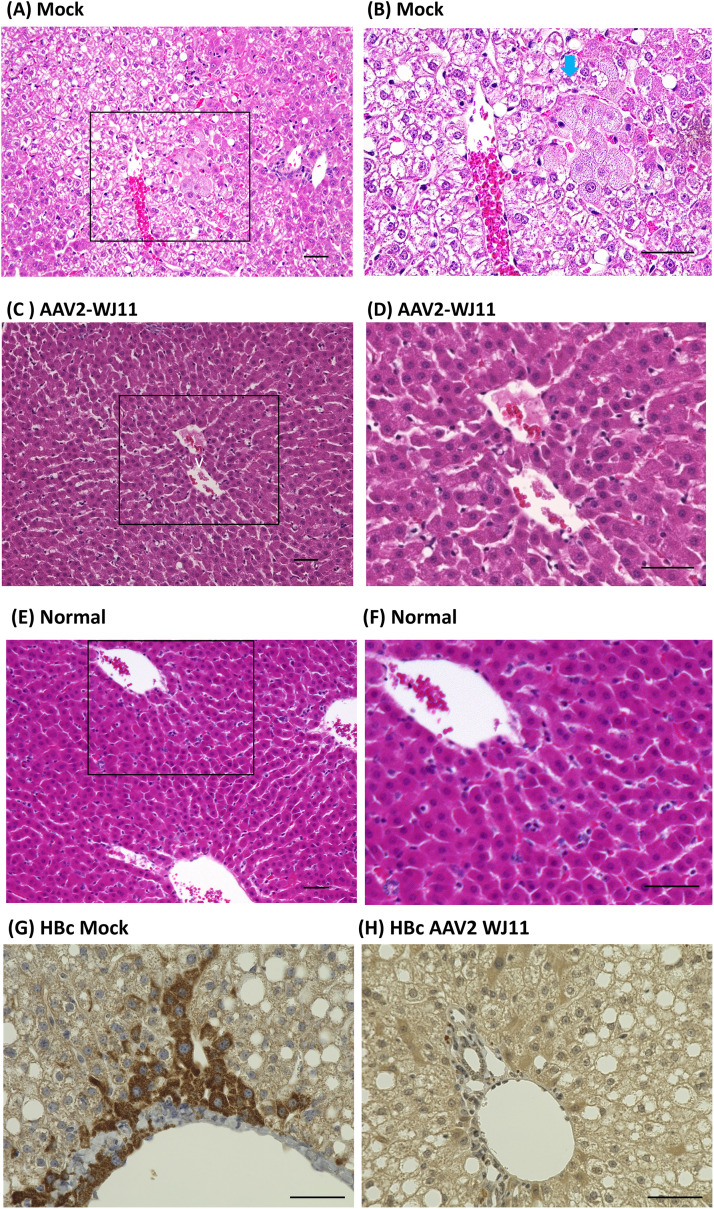
Histopathological analysis of liver tissues obtained from mock-treated control (A: ×200 and B: ×400, rectangle in A), AAV2/WJ11-Cas9 treated (C:x200 and D:x400, rectangle in C), and uninfected control (E:x200 and F:x400, rectangle in E). (A) Approximately 50 % to 60 % of the hepatocytes in the liver tissue showed balloon-like swelling due to hydropic degeneration. (B) The cytoplasm of some swollen hepatocytes was slightly eosinophilic and exhibited a ground-glass appearance (blue arrow). Histologically, no significant changes were observed in the liver of the AAV2/WJ11-Cas9-treated tupaia (C and D) and normal tupaia (E and F). Detection of HBcAg in mock-treated (G) and AAV2/WJ11-treated (H) tupaia liver. Bars indicate 50 μm.

Pathological analysis was performed on the liver 14 dpi (Fig. 3A–F). In mock-treated tupaias, 50 % to 60 % of the hepatocytes in the liver tissue exhibited balloon-like swelling due to hydropic degeneration (Fig. 3A). The cytoplasm of some swollen hepatocytes was slightly eosinophilic and exhibited a ground-glass appearance (Fig. 3B). Single-cell necrosis of the hepatocytes was not evident. Additionally, mild lymphocytic infiltration was observed in the interlobular connective tissues. In contrast, these abnormalities were absent in the livers of AAV2/WJ11-Cas9-treated tupaia (Fig. 3C and D) or normal tupaia livers (Fig. 3E and F). HBc antigen was detected in mock- (Fig. 3G) and AAV2/WJ11-Cas9-treated tupaia liver tissues (Fig. 3H), and a significant reduction of HBc antigen was observed in AAV2/WJ11-Cas9-treated tupaia liver tissues (Fig. 3H).

**Fig. 4:**
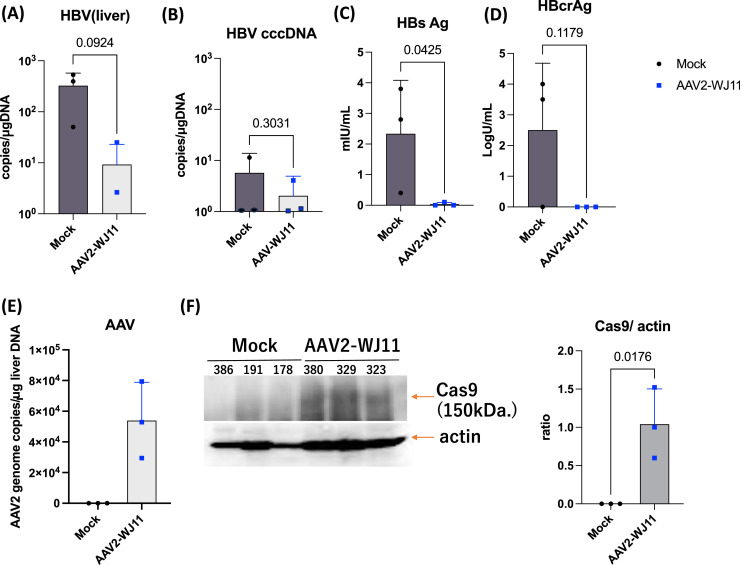
HBV DNA (A) and HBV cccDNA (B) levels in the liver of mock-treated (*n* = 3) and AAV2/WJ11-Cas9-treated tupaia (*n* = 3) at 14 dpi. Serum HBsAg (C) and HBcrAg (D) levels in mock (*n* = 3) and AAV2/WJ11-Cas9-treated tupaia (*n* = 3) at 14 dpi. In both groups, the levels of Anti-HBs and Anti-HBc antibodies increased, indicating that the tupaias were immunocompetent (E) AAV genome expression in tupaia liver was measured via qPCR. (F) Immunoblot assay of tupaia liver tissues lyzed with a lysis buffer at a concentration of 10 % weight per volume was performed using 10 μL to detected Cas9 protein and actin (left). Protein bands were quantitated and normalized using the Fusion chemiluminescence imaging system (right). Statistical significance was calculated using the Student's *t*-test and p-values are indicated. Error bars indicate the S.D. from three independent experiments.

**Fig. 5:**
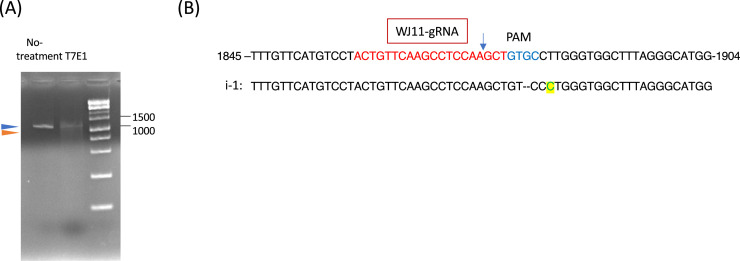
Mutagenesis effect of AAV2/WJ11-Cas9 in tupaia liver. (A) PCR of HBV genome was performed using Q5 polymerase with HBV-Fmt819F and 2027R primers (Kayesh et al., 2020) (left, 1.2 kb fragment, blue arrowhead) from AAV2/WJ11-Cas9-treated tupaia liver genome (#329). T7E1 assay was conducted using purified and amplified HBV 1.2 kb fragment (right, blue, and orange arrowhead). A 1 kb DNA ladder marker (Nippon Genetics Co.) was used as a DNA molecular weight marker. (B) Sequence of targeting region of AAV2/WJ11-Cas9. The amplified HBV genome (1.2 kb) from genomic DNA of the tupaia liver was subcloned and sequenced (i-1). The WJ11 gRNA sequence is shown in red, and the PAMs are indicated in blue. The blue arrow indicates the predicted Cas9 cutting sites (For interpretation of the references to color in this figure legend, the reader is referred to the web version of this article.).

We next examined whether AAV2/WJ11-Cas9 could effectively suppress HBV DNA and cccDNA in the liver (Fig. 4). AAV2/WJ11-Cas9 treatment significantly suppressed HBV DNA expression compared to the mock control (Fig. 4A). We also detected HBV cccDNA levels in liver genomic DNA, and a reduction was observed following AAV2/WJ11-Cas9 treatment (Fig. 4B). Serum HBsAg and HBcrAg levels were measured at 14 dpi, and a significant reduction was observed following AAV2/WJ11-Cas9 treatment (Fig. 4C and D). The presence of the AAV genome in liver tissue was confirmed through qPCR (5.4 × 10^4^ copies/μg genome DNA; Fig. 4E). Cas9 protein expression was detected in the AAV2/WJ11-Cas9 treated group (Fig. 4F); Cas9 protein was quantified and normalized to actin. The cleavage of the HBV genome by AAV2/WJ11-Cas9 was confirmed using the T7E1 assay (Fig. 5). HBV genome amplification via second-round PCR using the HBV-Fmt-819F and 2027R primers yielded the expected 1.2-kb fragment (Fig. 5A, blue arrow head) from genomic DNA purified from the liver tissue of tupaia 329. We performed a T7E1 assay on the HBV genome (1.2 kb) fragment, as described previously (Kayesh et al., 2020). The results showed digestion of AAV2/WJ11-Cas9-treated tupaia liver DNA, generating fragments of the expected size (*ca*. 1.0 kb, Fig. 5A, orange arrowhead), which appeared after T7E1 assay (Fig. 5A right). The amplified HBV genome (1.2 kb) was subcloned and sequenced (Fig. 5B). Indel mutations in the HBV genome generated in target regions by AAV/WJ11-Cas9 are shown in Fig. 5B (i-1).

### 3.3 Molecular analysis of mock- and AAV2/WJ11-Cas9-treated tupaia liver tissues

Given that AAV2/WJ11-Cas9-treated liver tissues showed significant recovery, we further characterized the modified host factors after AAV2/WJ11-Cas9 treatment. Consistent with the pathological analysis, the inflammatory cytokine IL-6 decreased to normal levels (Fig. S1). The level of TNF-α was relatively lower in AAV2/WJ11-Cas9-treated tupaia liver (Fig. S1). Additionally, the DNA sensor cyclic GMP-AMP (cGAMP) synthase, which suppresses HBV replication with STING (Kayesh et al., 2017a; Liu et al., 2015), was downregulated following AAV2/WJ11-Cas9 treatment. Conversely, IFN-β levels were increased after AAV2/WJ11-Cas9 treatment (Fig. S1). The level of IFN-g mRNA was highest in normal tupaia and decreased in mock-treated tupaia (Fig. S1).

**Fig. 6:**
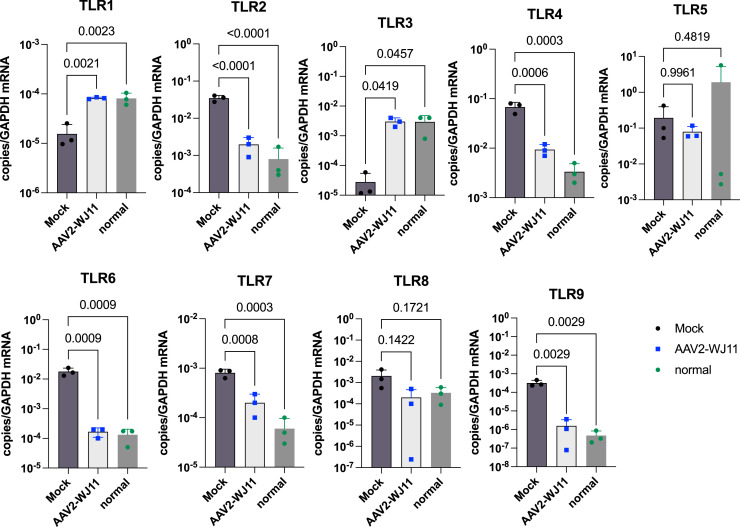
TLR levels in liver tissues of mock-treated (*n* = 3) and AAV2/WJ11-Cas9-treated (*n* = 3) tupaia at 14 dpi. Statistical significance was calculated using the Student's *t*-test, and p values are indicated. Error bars indicate the S.D. from three independent experiments.

To further clarify the modifications of upstream sensors due to HBV infection and the subsequent recovery induced by AAV2/WJ11-Cas9, we examined the mRNA expression levels of TLR1–9 (Fig. 6). Following HBV-F infection, *TLR2, TLR4, TLR6, TLR7,* and *TLR9* mRNAs were significantly upregulated and *TLR1* and *TLR4* were significantly downregulated in tupaia liver (Fig. 6). The modified mRNA expression levels of TLR1–9 induced by HBV-F infection returned to normal following AAV2/WJ11-Cas9 treatment, except for TLR5.

## 4 Discussion

The results of this study highlight the potential of WJ11/Cas9, delivered via AAV2 vectors, as a new therapeutic approach for suppressing HBV during the acute phase of infection in an immunocompetent model. Significant suppression of the viral load in serum HBV was observed in the AAV2/WJ11-Cas9-treated group at 1, 7, 10, and 14 dpi, compared with that in the mock-treated control group. Consistent with this observation, inflammation in liver tissues induced by HBV-F infection was significantly reduced following AAV2/WJ11-Cas9 treatment, including a reduction in hepatocyte necrosis. Among the inflammatory cytokines, IL-6 mRNA levels were significantly suppressed by AAV2/WJ11-Cas9. Elevated IL-6 mRNA levels are suggestive of more active hepatic necroinflammation and disease progression (Cressman et al., 1996; Kao et al., 2015, 2012). Therefore, AAV2/WJ11-Cas9 may have therapeutic effects against HBV-induced hepatitis. Furthermore, suppression of HBV infection was associated with increased levels of IFN-β, consistent with the previous findings that HBV suppresses IFN-β in tupaia (Kayesh et al., 2017a). The suppression of IFN-β by HBV may primarily result from the downregulation of *TLR3* which is a major regulator of IFN-β induction (Gao et al., 2021). The cyclic GMP-AMP synthase pathway, a key DNA sensor that suppresses HBV replication through STING, is downregulated following AAV2/WJ11-Cas9 treatment, potentially compromising the immune response and allowing HBV to evade detection more effectively (Verrier et al., 2018).

The expression of upstream TLRs was significantly modified by HBV-F but mostly returned to normal following AAV2/WJ11-Cas9 treatment, except for *TLR5*. Upregulation of *TLR2, TLR4, TLR6, TLR7,* and *TLR9* by HBV-F infection may contribute to the induction of inflammatory cytokine production (Kayesh et al., 2023), which was downregulated by AAV2/WJ11-Cas9 treatment. *TLR1* was significantly downregulated by HBV-F, which may reflect its role in suppressing inflammation (Kamdar et al., 2018). *TLR3* expression was also significantly suppressed by HBV-F, which likely contributed to the suppression of IFN-β induction, consistent with previous observations (Kayesh et al., 2017a). Thus, the expression of *TLR1–9*, except for *TLR5*, was shown to be closely linked with HBV-F in tupaia liver.

In our previous work, we showed the suppressive effect of AAV2/WJ11-Cas9 on HBV genotype C (Kayesh et al., 2020). In the present study, we show the suppressive effect of AAV2/WJ11-Cas9 on HBV-F. This effect may be attributed to the conserved nature of the gRNA sequence of WJ11 (nucleotide number 1859–1878) across HBV genotypes. The WJ11 gRNA sequence is perfectly conserved across HBV genotypes A–F, suggesting that AAV2/WJ11-Cas9 can be developed for pan-genotypic anti-HBV therapy. Although using the AAV2 vector alone as a negative control would have been more relevant to confirm the effect of AAV2/WJ11-Cas9 in targeting HBV, Cas9 expression and the detection of indel mutations in the HBV genome, as observed by the T7E1 assay, provide evidence of the direct effect of AAV2/WJ11-Cas9 on HBV genome disruption (Vouillot et al., 2015). In a previous study, the efficiency of target-specific mutagenesis of HBV DNA in mouse livers was approximately 11.0 %, as measured by deep sequencing in a HBV hydrodynamic injection- and CRISPR/Cas9-treated mouse model (Liu et al., 2015). Based on the results of this study, the mutagenesis rate was 3.8 %. Thus, increasing the amount of AAV2/WJ11-Cas9 may be necessary to achieve complete genome targeting in liver tissues during natural HBV infection. To further clarify the anti-HBV effect of AAV2/WJ11-Cas9, future studies should evaluate the effect of AAV vector alone on HBV DNA and cccDNA, although we already demonstrated a significantly higher effect of AAV2/WJ11-Cas9 compared to vector alone in HBV-infected humanized chimeric mice (Kayesh et al., 2020).

The results of this study indicated that the tupaia HBV infection model is a useful tool for characterizing host immune responses (Sanada et al., 2019; Tsukiyama-Kohara and Kohara, 2014; Yan et al., 2012). To evaluate AAV2/WJ11-Cas9, we used 5 × 10^12^ copies for each animal, and transduction was confirmed via AAV detection in the liver tissues. Considering the body weight and liver size of tupaia (average body weight 150 g, average liver weight 10 g), it is estimated that at least 10^14–15^ copies of AAV2/WJ11-Cas9 would be required for a human patient (body weight, 60 kg, liver 1–1.5 kg). This strongly suggests that improving delivery systems is essential to facilitate the application of CRISPR/Cas9 technology in human gene therapy. AAV vectors, particularly AAV2, are widely regarded as highly effective delivery vehicles for CRISPR-Cas9 components *in vivo* due to their high transduction efficiency and low immunogenicity. However, AAV8, a hepatotropic variant, could serve as a promising candidate for further optimization, contributing enhanced targeting specificity for liver cells and potentially improving the efficacy of gene editing in liver-associated diseases (Li et al., 2018; Wang et al., 2024; Yan et al., 2021).

Our findings enhance our understanding of the immune response of immunocompetent HBV model animals in the acute phase of HBV infection, which could further advance translational medicine. However, this study had limitations that should be addressed. We focused on the acute phase of HBV infection at 14 dpi, which means that our results may not be fully generalizable to long-term or chronic treatment outcomes. Furthermore, the tupaia model, while valuable for HBV research, may not fully replicate human immune responses, thus limiting direct applicability. Certain markers, such as HBeAg or Anti-HBe antibody, were undetectable within the study timeframe, potentially missing important early immune responses. In addition, cytokine analysis was conducted solely at 14 dpi, potentially overlooking important immune response changes at later stages. These limitations underscore the importance of future research with an extended study duration, varied animal models, and broader immune response analyses to enhance the understanding and applicability of the findings.

To our knowledge, this is the first study to evaluate the CRISPR/Cas9 system in an immunocompetent HBV infection model. Overall, our findings suggest that WJ11-Cas9 delivered using AAV2 vectors may represent a new therapeutic strategy for eliminating HBV infection. Additionally, this study provides valuable molecular insights into HBV-induced inflammation. However, chronic HBV infection remains a major global health problem due to the persistence of cccDNA and integrated HBV DNA replicative templates. Moreover, HBV mutations can occur during reverse transcription at an estimated rate of 1–3 × 10^−5^ nucleotide substitutions per site per year, increasing the risk of resistance to nucleoside/nucleotide analog therapies. Consequently, alternative therapeutic approaches are required to treat effectively HBV infections. Genome editing enables the manipulation of target genes in various cell types using engineered nucleases. Compared with ZFNs and TALENS, the CRISPR/Cas9 system can be easily reprogrammed to cleave virtually any DNA sequence by redesigning native or engineered crRNAs. Although CRISPR/Cas9 has revolutionized gene editing, viral delivery in immunocompetent animals has not been thoroughly explored. Furthermore, evaluating CRISPR/Cas9 in an HBV-infected tupaia model could provide a reference for future therapeutic interventions in translational medicine. Accordingly, further studies are warranted to investigate the efficacy of the CRISPR/Cas9 therapeutic strategy for treating chronic HBV infection.

## Glossary

CRISPR/Cas9: Gene editing method using Cas9 enzymes that recognize guide RNA sequences

ZFN (zinc finger nuclease): The first genome editing tool that recognizes FokI digestion site.

TALEN: Transcription activator-like effector nuclease composed of TALE protein and FokI nuclease.

## Funding sources

This work was supported by grants from the Japan Agency for Medical Research and Development (K07S411687S, 23fk0310515h0002, and 24fk0310515h0003) and the Ministry of Health, Labour, and Welfare of Japan.

## CRediT authorship contribution statement

**Md Haroon Or Rashid:** Writing – review & editing, Writing – original draft, Data curation. **Mohammad Enamul Hoque Kayesh:** Writing – review & editing, Writing – original draft, Validation, Data curation. **Md Abul Hashem:** Writing – review & editing, Data curation, Conceptualization. **Tatsuro Hifumi:** Writing – review & editing, Data curation. **Shintaro Ogawa:** Methodology, Data curation. **Noriaki Miyoshi:** Methodology, Data curation. **Yasuhito Tanaka:** Methodology, Data curation. **Michinori Kohara:** Writing – review & editing, Writing – original draft, Supervision, Data curation, Conceptualization. **Kyoko Tsukiyama-Kohara:** Writing – review & editing, Writing – original draft, Funding acquisition, Data curation, Conceptualization.

## Declaration of competing interest

The authors declare no conflict of interest.

## Data Availability

Data will be made available on request.

## Appendix Supplementary materials

Image, application 1
