# Supplementary material for: Adeno-associated virus 2 CRISPR/Cas9-mediated targeting of hepatitis B virus in tree shrews
Source: Virus Res. 2025 Feb 21;354:199550. doi: 10.1016/j.virusres.2025.199550 (PMC11909760; doi:10.1016/j.virusres.2025.199550)
Supplement: Supplementary file 1 [file mmc1.docx]

**Supplementary information**

**Adeno-associated virus 2 CRISPR/Cas9-mediated targeting of hepatitis B virus in tree shrew**

Md Haroon Or Rashid^a^, Mohammad Enamul Hoque Kayesh^a,b,c^, Md Abul Hashem^a, b, d^, Tatsuro Hifumi^b,e^, Shintaro Ogawa^f^, Noriaki Miyoshi^e^, Yasuhito Tanaka^f^, Michinori Kohara^g^, Kyoko Tsukiyama-Kohara^a,b#^

^a^Laboratory of Animal Hygiene, Joint Faculty of Veterinary Medicine, Kagoshima University, Kagoshima, Japan.

^b^Transboundary Animal Diseases Centre, Joint Faculty of Veterinary Medicine, Kagoshima University, Kagoshima, Japan.

^c^Department of Microbiology and Public Health, Patuakhali Science and Technology University, Bangladesh.

^d^Department of Cell and Developmental Biology, Feinberg School of Medicine, Northwestern University, Chicago, IL, USA

^e^Department of Veterinary Histopathology, Joint Faculty of Veterinary Medicine, Kagoshima University, Kagoshima, Japan.

^f^Faculty of Life Sciences, Kumamoto University 1-1-1 Honjo, Chuo-ku, Kumamoto

^g^Department of Microbiology and Cell Biology, Tokyo Metropolitan Institute of Medical Science, Japan.

**Table S1. Primer sequences used in quantitative reverse transcription-polymerase chain reaction.**

| **Gene** | **Primer sequences (5′-3′ )**  **Forward (F), Reverse (R)** | **Product length (bp)** | **Reference** |
| --- | --- | --- | --- |
| *tGAPDH* | F: AATTTGGCTACAGCAACAGG | 234 | Kayesh et al., 2017a |
|  | R: ATTGATGGTTCGTGACAAGG |  |  |
| *tActin* | F: GAGCATCCCTAGAGTTCTGCAA | 102 | Kayesh et al., 2017a |
|  | R: TCCTGTAACAATGCGTCTCACA |  |  |
| *tTLR1* | F: TGCTGACTGTGACCATGACC | 105 | Kayesh et al., 2017a |
|  | R: GCAAGTTCCTTGCTCTGCG |  |  |
| *tTLR2* | F: AGCTGCTGTTTTACGCTT | 160 | Kayesh et al., 2017a |
|  | R: AGGTAAAACTTGGGGATGTG |  |  |
| *tTLR3* | F: AGCCTTCAACGACTGATGCT | 264 | Kayesh et al., 2017a |
|  | R: GTTGAGGACGTGGAGGTGAT |  |  |
| *tTLR4* | F: TACAGAAGCTGGTGGCTGTG | 152 | Kayesh et al., 2017a |
|  | R: CTCCAGGTTGGGCAGGTTAG |  |  |
| *tTLR5* | F: GCTGGTCAGTGGACATCACA | 147 | Kayesh et al., 2017a |
|  | R: CCAGGCCAGCAAATGTGTTC |  |  |
| *tTLR6* | F: GTGGAGGACTGGCCTGATTC | 168 | Kayesh et al., 2017a |
|  | R: GATGCAGAGGAGGGTCATGG |  |  |
| *tTLR7* | F: AGATGTCCCCACTGTTTTGC | 141 | Kayesh et al., 2017a |
|  | R: TAACAACGAGGGCAGTTTCC |  |  |
| *tTLR8* | F: AAACCTCTCTAGCACTTC | 152 | Kayesh et al., 2017a |
|  | R: CAAGTGTTTCTAAGTAGTCC |  |  |
| *tTLR9* | F: TATAACTGCATCGCGCAGAC | 257 | Kayesh et al., 2017a |
|  | R: CGGCTGTGGATATTGTTGTG |  |  |
| *tIFN-β* | F: GCAGCAGTTTGGCGTGTAAG | 121 | Kayesh et al., 2017a |
|  | R: TTCTGGAACTGCTGTGGTCG |  |  |
| *tIFN-γ* | F: TACACTGGCTTTCCTGCTTTCTATC | 179 | *Kayesh et al., 2022a |
|  | R: TTTTGTCACTCTCCTCTGTCCAA |  |  |
| *tIL-6* | F: ATACCAGAACCCACCTCCAC | 115 | Kayesh et al., 2017a |
|  | R: GTGCAACCCTGCACTTGTAA |  |  |
| *tTNF-α* | F: GCCTAGTCAACCCTCTGACC | 100 | Kayesh et al., 2017a |
|  | R: CCCTTGTTTTGGGGGTTTGC |  |  |
| *tcGAS* | F: ACGCAAAGGAAGGAAGTGGT | 145 | *Kayesh et al., 2017a |
|  | R: TTTAAACAATCTTTCCTGCAACA |  |  |

*Kayesh MEH, Hashem MA, Sanada T, Kitab B, Rashid MHO, Akter L, Ezzikouri S, Murakami S, Ogawa S, Tanaka Y, Kohara M, Tsukiyama-Kohara K. 2022. Characterization of innate immune response to hepatitis B virus genotype F acute infection in tree shrew (*Tupaia belangeri*) model. Front Virol 2. DOI [10.3389/fviro.2022.926831](https://doi.org/10.3389/fviro.2022.926831).

**Fig. S1**. Cytokine levels in liver tissues of mock-treated (n = 3) and AAV2/WJ11-Cas9-treated (n = 3) tupaia at 14 dpi. Statistical significance was calculated using the Student’s *t*-test, and p values are indicated. Error bars indicate the S.D. from three independent experiments.
